# Shaping of CD56^bri^ Natural Killer Cells in Patients With Steroid-Refractory/Resistant Acute Graft-vs.-Host Disease via Extracorporeal Photopheresis

**DOI:** 10.3389/fimmu.2019.00547

**Published:** 2019-03-20

**Authors:** Ming Ni, Lei Wang, Mingya Yang, Brigitte Neuber, Leopold Sellner, Angela Hückelhoven-Krauss, Maria-Luisa Schubert, Thomas Luft, Ute Hegenbart, Stefan Schönland, Patrick Wuchter, Bao-an Chen, Volker Eckstein, William Krüger, Ronit Yerushalmi, Katia Beider, Arnon Nagler, Carsten Müller-Tidow, Peter Dreger, Michael Schmitt, Anita Schmitt

**Affiliations:** ^1^Department of Internal Medicine V, University Clinic Heidelberg, Heidelberg, Germany; ^2^Department of Hematology, the Affiliated Hospital of Guizhou Medical University, Guiyang, China; ^3^German Red Cross Blood Service Baden-Wuerttemberg-Hessen, Institute of Transfusion Medicine and Immunology, Medical Faculty Mannheim, Heidelberg University, Mannheim, Germany; ^4^Department of Hematology, Zhongda Hospital, Southeast University, Nanjing, China; ^5^Department of Internal Medicine C, Hematology, Oncology, Stem Cell Transplantation, Palliative Care, University Clinic Greifswald, Greifswald, Germany; ^6^Hematology Division, Chaim Sheba Medical Center, Tel Hashomer, Ramat Gan, Israel

**Keywords:** GvHD, ECP, immunomodulation, natural killer cells, anti-viral effect, anti-leukemia effect

## Abstract

CD56^bri^ natural killer (NK) cells play an important role in the pathogenesis of graft-vs. -host disease (GVHD) and immune defense in the early period after allogeneic hematopoietic stem cell transplantation. Extracorporeal photopheresis (ECP) as an immunomodulating therapy has been widely used for GVHD treatment. However, the mechanism of action of ECP still remains to be elucidated, particularly the influence of ECP on NK cells. Thirty-four patients with steroid-refractory/resistant acute GVHD (aGVHD) ≥ °II and moderate to severe chronic GVHD (cGVHD) received ECP therapy. Patient samples obtained during intensive and long-term treatment were analyzed. Immunomonitoring with respect to cell phenotype and function was performed on rested peripheral blood mononuclear cells (PBMCs) using multiparametric flow cytometry. NK activity in terms of cytokine release was analyzed by intracellular cytokine staining after co-culture with K562 cells. Moreover, the proliferative capacity of NK cells, CD4^+^, and CD8^+^ T cells was determined by carboxyfluorescein succinimidyl ester (CFSE) staining. Clinically, 75% of aGVHD and 78% of cGVHD patients responded to ECP therapy. Moreover, our data show that aGVHD, cGVHD patients and healthy donors (HDs) present distinct NK patterns: aGVHD patients have a higher frequency of CD56^bri^ NK subsets with stronger NKG2D and CD62L expression, while CD56^−^CD16^+^ NK cells with higher expression of CD57 and CD11b stand out as a signature population for cGVHD. ECP therapy could significantly decrease CD56^bri^CD16^−^ NK cells with shifting the quality from a cytotoxic to a regulatory pattern and additionally mature CD56^dim^ NK cells via upregulation of CD57 in complete responding aGVHD patients. Moreover, ECP could keep the anti-viral and anti-leukemic effects intact via maintaining specialized anti-viral/leukemic CD57^+^NKG2C^+^CD56^dim^ NK cells as well as remaining the quality and quantity of cytokine release by NK cells. The proliferative capacity of effector cells remained constant over ECP therapy. In conclusion, ECP represents an attractive option to treat GVHD without compromising anti-viral/leukemic effects. Shaping of CD56^bri^ NK cell compartment by downregulating the cytotoxic subset while upregulating the regulatory subset contributes to the mechanisms of ECP therapy in aGVHD.

## Introduction

Extracorporeal photopheresis (ECP) is being widely used for the treatment of T cell-mediated diseases e.g., graft-vs.-host disease (GVHD) with established clinical benefits (1). ECP therapy can rebalance the destroyed immune system in the case of GVHD by (a) direct induction of alloreactive T cell apoptosis, (b) downregulation of proinflammatory cytokines, (c) selective modulation of trafficking patterns of alloreactive T cells, and (d) increase of different regulatory cells such as CD4^+^ and CD8^+^ regulatory T cells, regulatory B cells, and myeloid-derived suppressor cells (2–5). Nevertheless, the mechanism of action of ECP still needs to be further elucidated, particularly with regard to natural killer (NK) cells.

CD56^bri^ NK cells are important innate immune cells that are the first lymphocyte subset which reconstitutes after allogeneic hematopoietic stem cell transplantation (allo-HSCT) and as such provides a temporal bridge of protection from opportunistic infections and prevention of cancer relapse during the transient period of T-cell deficiency post-transplantation (6–8). Therefore, the early rapid reconstitution of CD56^bri^ NK cells is of crucial importance for the post-transplantation outcomes. This has been reported by several studies documenting CD56^bri^ NK cells in correlation to a better survival and less transplantation-related mortality (9–12). In addition, clinical data illustrated that a low frequency of CD56^bri^ NK cells is associated with the development of GVHD (12, 13). This theory was further confirmed by a recent study showing that ECP could reduce GVHD by upregulating CD56^bri^ NK cells (14). However, since CD56^bri^ NK cells are the most efficient cytokine producers (15), theoretically they can also contribute to the induction and exacerbation of GVHD through releasing of proinflammatory cytokines like interferon-γ (IFN-γ) and tumor necrosis factor (TNF-α) (16–18). The question arises therefore how and to which extent ECP induced CD56^bri^ NK cells might contribute to the control of GVHD and this is the aim of our current study.

## Subjects, Materials, and Methods

### Subjects

Thirty-four patients suffering from steroid-refractory/resistant acute GVHD (aGVHD) ≥ °II and moderate to severe chronic GVHD (cGVHD) from the University Hospitals Heidelberg and Greifswald in Germany as well as Chaim Sheba Medical Center in Israel were included. The study was approved by the Institutional Review Boards. Written informed consent was obtained from all patients.

### ECP Treatment

ECP therapy was performed with a Therakos UVAR XTS^®^ and a CELLEX^®^ Photopheresis System involving *ex vivo* exposure of leukapheresed peripheral blood mononuclear cells (PBMCs) to ultraviolet-A light in the presence of 8-methoxypsoralen (8-MOP) and reinfusion of the treated cells to patients. aGVHD patients received intensive semiweekly treatment in the first 12 weeks, followed by biweekly treatment. cGVHD patients received either semiweekly treatment followed by a biweekly treatment or a biweekly treatment *upfront*. ECP therapy was administered until reaching the best response.

### Clinical Evaluation

Clinical assessment of aGVHD and cGVHD was undertaken according to the current guidelines (19–21). Response to ECP treatment was defined as complete response (CR), partial response (PR), stable disease (SD), and no response (NR). CR was defined as the resolution of all reversible manifestations. PR was defined as the improvement of clinical symptoms of the involved organ concomitant with a reduction of steroid dose. SD was defined as the reduction of steroid dose with slight improvement of clinical symptoms. NR was defined as the absence of improvement of clinical symptoms.

### Cell Preparation

Peripheral blood was drawn from the patients before the ECP treatment. Buffy coats from consenting healthy donors (HDs) were obtained from the Heidelberg blood bank after overnight storage. PBMCs were isolated by gradient centrifugation followed by washing twice with phosphate buffered saline (PBS) (Sigma). Afterwards, cells were stored in liquid nitrogen till immunomonitoring. A standard resting process was performed to restore the function and the antigenic expression of cells as previously described (22). Briefly, cells were resuspended at 2 × 10^6^ cells/ml with complete medium [CM: RPMI medium 1640 (Gibco) containing 10% fetal bovine serum (FBS) (Sigma)] after thawing. Afterwards cells were rested at 37°C, 5% CO_2_ for 18 hours in a horizontal position.

### Cell Line

K562, a highly undifferentiated human erythroleukemic cell line, was maintained in CM at 37°C, 5% CO_2_. Medium was changed every 3 days to passage cells. Mycoplasma contamination was checked by polymerase chain reaction (PCR) before each experiment.

### Stimulation of NK Cells

5 × 10^5^ rested PBMCs were co-cultured with 5 × 10^4^ K562 cells in the presence of CD107a antibody at 37°C, 5% CO_2_ in a 96-U-bottom plate for 6 hours. After the first hour incubation, 1 μl of 100X monensin (Biolegend) and Brefeldin A (Biolegend) were added into each well. PBMCs alone group was served as negative control.

### Multiparameter Flow Cytometry

The quality and quantity of expression of different markers were determined by multiparametric flow cytometry. Samples were stained by different combinations of antibodies against CD3, CD4, CD8, CD11b, CD14, CD16, CD19, CD27, CD56, CD57, CD62L, CD107a, CD159c (NKG2C), CD314 (NKG2D), IFN-γ, and TNF-α. Detailed information of antibody is shown in Supplementary Table 1. 7-Amino actinomycin D (7AAD) or Near-infrared (NEAR-IR) was used for live/dead cell discrimination. Fluorescence minus one, unstimulated, and autofluorescence controls were included in order to place the gate more accurately. To reduce the variation, samples from the same patient at different time points have been analyzed on the same day. The Fc receptors were blocked by blocking buffer A [50% fluorescence-activated cell sorting buffer (FACS) + 50% human serum] or blocking buffer B (50% perm buffer + 50% human serum) prior to surface marker staining or intracellular cytokine staining, respectively. All acquisitions were performed on a LSRII device (BD Biosciences) and the data were analyzed by FACS Diva software (BD Biosciences). The cellular division index was determined by Flowjo software (TreeStar).

#### Surface Marker Staining

After 10 min blocking at 4°C, 5 × 10^5^ rested PBMCs were stained with different antibodies for 20 min at 4°C in the dark.

#### Intracellular Cytokine Staining

Briefly, 5 × 10^5^ rested cells were stained with NEAR-IR for 30 min at 4°C in the dark. Thereafter, cells were stained with surface marker antibodies, followed by fixation and permeabilization according to the Miltenyi Foxp3 fix/perm buffer instruction. A 15 min blocking step was performed prior to the intracellular antibody staining [30 min, room temperature (RT)].

### Assessment of Proliferation Function

Freshly thawed PBMCs were washed and resuspended with 1 ml PBS containing 5% FBS in 15 ml tubes. 1 μl of 5 mM carboxyfluorescein succinimidyl ester (CFSE) solution (Biolegend) was directly added into the cell suspension followed by 5 min incubation at RT in the dark. The reaction was stopped by adding 5 ml cold CM and cells were washed twice with 5 ml CM. After staining, 2 × 10^5^ CFSE-labeled cells were seeded into each well and stimulated with either 100 ng/ml interleukin-15 (IL-15) (R&D systems) or 1 μg/ml staphylococcal enterotoxin B (SEB) (Sigma) for NK cells and T cells, respectively. Cell proliferation was analyzed after 3 and 7 days culture for NK cells and T cells, respectively.

### Statistical Analysis

Analysis was undertaken using SPSS version 24 (IBM) for windows software. One-way ANOVA with Bonferroni *post-hoc* test was performed to assess the differences of the marker expression and the cytokine release pattern among HDs, patients with aGVHD and cGVHD within the five different NK subsets. Differences between two different time points and two different groups were determined by Wilcoxon signed-rank test and Mann-Whitney U test, respectively. A *p*-value < 0.05 was considered to be statistically significant.

## Results

### Demographics and Clinical Response

Patient characteristics and clinical response to ECP therapy are summarized in Tables 1, 2. Sixteen patients with steroid-refectory/resistant aGVHD (6 men and 10 women aged 23–68 years) and 18 patients with cGVHD (11 men and 7 women aged 32–70 years) were treated by ECP. The median time from GVHD onset to commencing ECP was 33.5 days for aGVHD (range: 7–373 days) and 363.5 days for cGVHD (range: 14–4240 days). The median number of ECP cycles was 11 (range: 5–34 cycles) and 37 (range: 4–90 cycles) for aGVHD and cGVHD patients, respectively. 75% (12/16) of the patients with aGVHD and 78% (14/18) of those with cGVHD responded to the ECP treatment. Only two patients with aGVHD had cytomegalovirus (CMV) reactivation after the first 2–3 ECP treatment while still on 2 mg/kg per body weight steroids. As control, 10 healthy donors (5 men and 5 women aged 20–66 years) have been analyzed in our study. Three of them were tested positive for CMV.

**Table 1:** aGVHD patients' characteristics and clinical response to ECP.

**# Pat**.	**Primary disease**	**Type of transplantation**	**Stem cell source**	**CMV status D/R**	**Prophylaxis for GVHD**	**aGVHD grade**	**Organ involved**	**CMV reactivation**	**ECP response**
1	CLL	MUD	PBSC	–/–	ATG+MMF+TAC	°III	gut	–	ST
2	FL	MUD	PBSC	–/–	ATG+MMF+TAC	°III-IV	gut	–	CR
3	AML	MMUD	PBSC	–/–	ATG+MMF+TAC	°III	gut	–	PR
4	AML	Haplo	PBSC	–/+	MMF+TAC	°III	gut	–	ST
5	CLL	MUD	PBSC	+/+	ATG+MMF+TAC	°III	gut	–	NR
6	TPLL	MMUD	PBSC	–/–	ATG+MMF+TAC	°III	gut	–	NR
7	CML	MMUD	PBSC	–/–	ATG+CsA+MMF	°II	gut	–	CR
8	AML	MUD	PBSC	–/–	ATG+MMF+TAC	°III	gut	–	CR
9	AML	MRD	PBSC	+/+	CsA+MMF	°II	gut	–	PR
10	CLL	MUD	PBSC	–/–	ATG+MMF	°III	gut	–	PR
11	FL	MRD	PBSC	–/+	CsA+MMF	°IV	gut	+^*^	PR
12	MDS	MRD	PBSC	+/+	MMF+TAC	°III/°IV	gut, skin	+^*^	PR
13	AML	MUD	PBSC	–/–	ATG+CsA+MMF	°III	gut	–	PR
14	AML	MRD	PBSC	+/+	CsA+MMF	°III	gut	–	PR
15	CMML	MRD	PBSC	+/+	CsA+MMF	°III /°IV	gut	–	CR
16	CTCL	MUD	PBSC	+/+	ATG+TAC	°III	gut	–	PR

aGVHD, acute GVHD; ECP, Extracorporeal photopheresis; # Pat., Patient number; CLL, Chronic Lymphocytic Leukemia; FL, Folicular Lymphoma; AML, Acute Myeloid Leukemia; TPLL, T cell Prolymphocytic Leukemia; CML, Chronic Myelogenous Leukemia; MDS, Myelodysplastic Syndromes; CMML, Chronic Myelomonocytic Leukemia; CTCL, Cutaneous T cell Lymphoma; MUD, Matched unrelated donor; MMUD, Mismatched unrelated donor; Haplo, Haploidentical Stem Cell Transplantation; MRD, matched related donor; PBSC, Peripheral blood stem cell; CMV, Cytomegalovirus; D, Donor; R, Recipient; –, negative; +, positive; ATG, Anti-thymocyte globulin; MMF, Mycophenolate mofetil; TAC, Tacrolimus; CsA, Cyclosporine A; ST, Short treatment; CR, Complete response; PR, Partial response; NR, No response;

* *means after the first 2–3 ECP treatment still under 2 mg/kg body weight steroids*.

**Table 2:** cGVHD patients' characteristics and clinical response to ECP.

**# Pat**.	**Primary disease**	**Type of transplantation**	**Stem cell source**	**CMV status D/R**	**Prophylaxis for GVHD**	**cGVHD grade**	**Organ involved**	**CMV reactivation**	**ECP response**
17	LL	MRD	PBSC	+/–	CsA+MTX	moderate	skin	–	PR
18	DLBCL	MMUD	PBSC	–/–	ATG+CsA	severe	lung	–	SD
19	AML	MRD	BM	+/+	CsA	severe	lung	–	PR^*^
20	AILT	MRD	PBSC	+/–	MMF	moderate	skin	–	SD
21	TPLL	MUD	PBSC	+/+	ATG+TAC	moderate	skin	–	PR
22	AML	MRD	PBSC	+/-	MMF+SRL	severe	skin	–	ST
23	PTCL	MUD	PBSC	+/–	ATG+CsA+MMF	moderate	skin	–	CR
24	CLL	MRD	PBSC	+/+	MMF	moderate	skin	–	PR
25	TPLL	Haplo	BM	–/–	MMF+TAC	severe	skin	–	ST
26	OMF	MUD	PBSC	+/+	MMF+TAC	severe	skin	–	PR
27	CLL	MUD	PBSC	–/–	CsA+MMF	severe	skin	–	PR^*^
28	ALL	MRD	PBSC	+/+	MMF	severe	skin	–	SD
29	HL	MRD	PBSC	+/+	CsA+MMF	severe	skin	–	PR
30	BAL	MRD	PBSC	–/–	CsA	severe	extent	–	SD
31	AML	MRD	PBSC	+/+	CsA	moderate	skin	–	PR
32	AML	MUD	PBSC	–/–	ATG+CsA+MMF	severe	skin	–	SD
33	MDS	MRD	PBSC	+/+	PDN	severe	skin	–	PR
34	MCL	MRD	PBSC	–/–	CsA+MMF	severe	skin	–	PR

cGVHD, chronic GVHD; ECP, Extracorporeal photopheresis; # Pat., patient number; LL, Lymphoblastic Lymphoma; DLBCL, Diffuse Large B cell Lymphoma; AML, Acute Myeloid Leukemia; AILT, Angioimmunoblastic T cell Lymphoma; TPLL, T Prolymphocytes Leukemia; PTCL, Peripheral T cell Lymphoma; CLL, Chronic Lymphocytic Leukemia; OMF, Osteomyelofibrosis; ALL, Acute Lymphoblastic Leukemia; HL, Hodgkin's lymphoma; BAL, Biphenotypic Acute Leukemia; MDS, Myelodysplastic Syndromes; MCL, Mantle cell Lymphoma; MUD, mismatched unrelated donor; MMUD, matched unrelated donor; MRD, matched related donor; Haplo, Haploidentical Stem Cell Transplantation; PBSC, Peripheral blood stem cell; BM, Bone marrow; CMV, Cytomegalovirus; D, Donor; R, Recipient; –, negative; +, positive; CsA, Cyclosporine A; MTX, Methotrexate; ATG, Anti-thymocyte globulin; MMF, Mycophenolate mofetil; TAC, Tacrolimus; SRL, Sirolimus; PDN, Prednisolon; PR, Partial response; SD, Stable disease; PR

* *, Partial response but progression afterward; ST, Short treatment; NR, No response; CR, Complete response*.

### Distinct NK Cells Pattern

**Figure 1:**
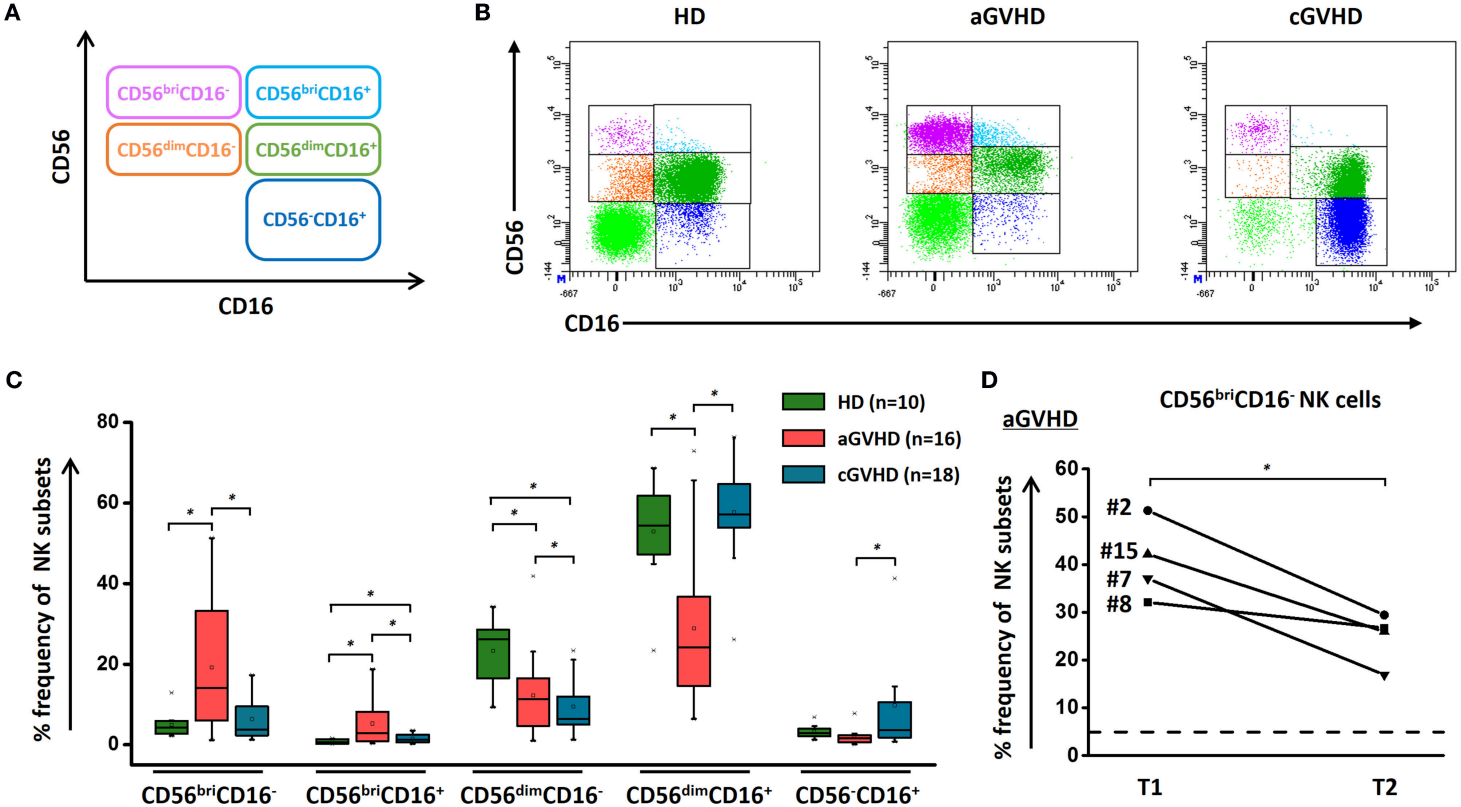
NK subsets in GVHD and effect of ECP on CD56^bri^ NK subset. NK cells could be defined as five different subsets by relative expression of CD56 and CD16 **(A)**. The representative dot plots **(B)** and box plots **(C)** show that the component of NK cells is different among aGVHD patients (*n* = 16), cGVHD patients (*n* = 18), and HDs (*n* = 10). ECP could dramatically decrease the frequency of CD56^bri^CD16^−^ NK cells in aGVHD patients with CR (*n* = 4) **(D)**. Dashed line represents the corresponding median value of frequencies observed in 10 healthy donors. ^*^p < 0.05.

Based on CD56 and CD16 expression, we could define five different NK cell subsets, as shown in Figure 1A. Of note, HDs, patients with aGVHD and cGVHD displayed distinct patterns of these five different subsets. Figure 1B depicts representative dot plots among HDs, aGVHD, and cGVHD patients. Patients suffering from steroid-refractory/resistant aGVHD were characterized by a higher frequency of CD56^bri^ NK cells when compared with HDs and patients with cGVHD (Figure 1C). Moreover, this signature population of aGVHD, CD56^bri^CD16^−^ NK cells, can be significantly downregulated by ECP treatment in patients achieving complete response (Figure 1D) but not in patients with PR and NR (Supplementary Figure 1). In contrast, CD56^dim^CD16^+^ and CD56^−^CD16^+^ NK cell subsets were prominent in patients with cGVHD (Figure 1C). Especially, a significant increase of CD56^−^CD16^+^ NK cells was observed in the cGVHD cohort.

**Figure 2:**
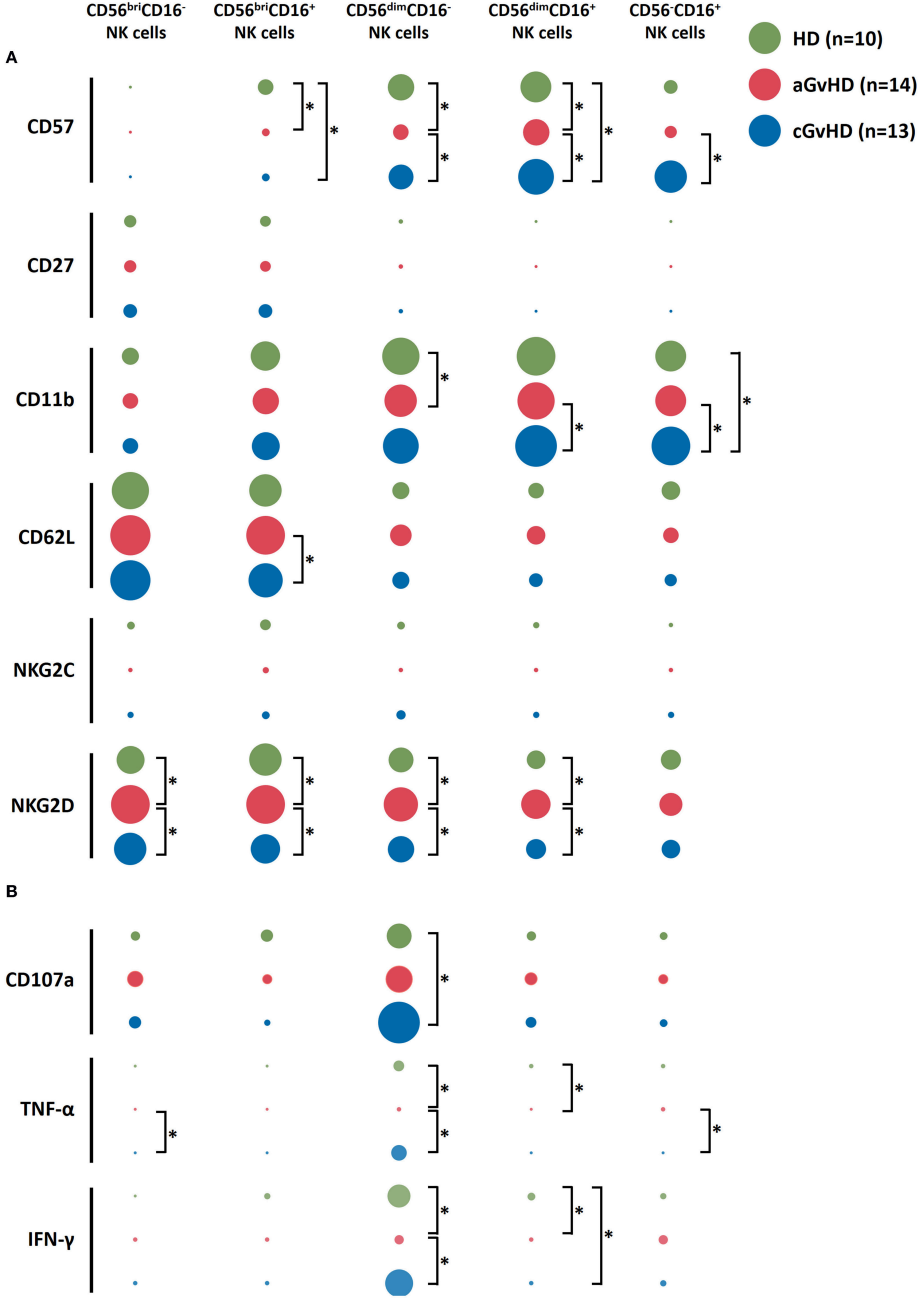
Characterization of NK subsets. NK subsets display not a distinct immunophenotype based on the surface markers expression **(A)** but also a different functional profile upon K562 stimulation **(B)** among aGVHD (*n* = 14, excluding patient #5 and #8 due to the limited cell number of samples), cGVHD (*n* = 13, excluding patient #17, #18, #21, #22, and #25 due to the limited cell number of samples) and HD groups (*n* = 10). The figure was drawn by Excel software with “EasyCharts” package. The data were normalized automatically by the software. The diameter of the bubble presents the mean value of the percentage of the expression of each marker. ^*^p < 0.05.

To further characterize these different NK cell subsets, the expression of cell surface markers and cytokine profile upon K562 stimulation were examined (Figure 2). In patients with aGVHD at baseline pre-ECP treatment, we observed a decreased expression of the maturation markers CD57 and CD11b on NK cell subsets (Figure 2A). By contrast, significantly higher expression of these maturation markers was detected on NK cell subpopulations in patients with cGVHD when compared to HDs and patients with aGVHD (Figure 2A). Furthermore, we observed a significant elevation of the NK activation marker NKG2D on NK cells in patients with aGVHD. In addition, the immature markers CD27 and CD62L as well as the CMV specific activating receptor NKG2C display a similar expression on these five different NK subsets among the HDs, aGVHD and cGVHD groups with exception of CD56^bri^CD16^+^ NK cells that showed a high expression of CD62L in aGVHD patients.

Besides surface marker expression, the anti-tumor function of the NK subsets upon K562 stimulation was evaluated (Figure 2B). CD56^dim^CD16^−^ subset showed the highest level of CD107a expression as well as the biggest amount of TNF-α and IFN-γ secretion compared to other subpopulations, suggesting their crucial role in the anti-tumor capacity of the NK cells. Interestingly, although the NK cells had less potency to secrete cytokines in aGVHD patients, a stronger CD107a expression on CD56^bri^CD16^−^ NK cells could be induced by co-culturing with K562 cells (Figure 2B).

### Maturation of NK Cells by ECP Therapy

**Figure 3:**
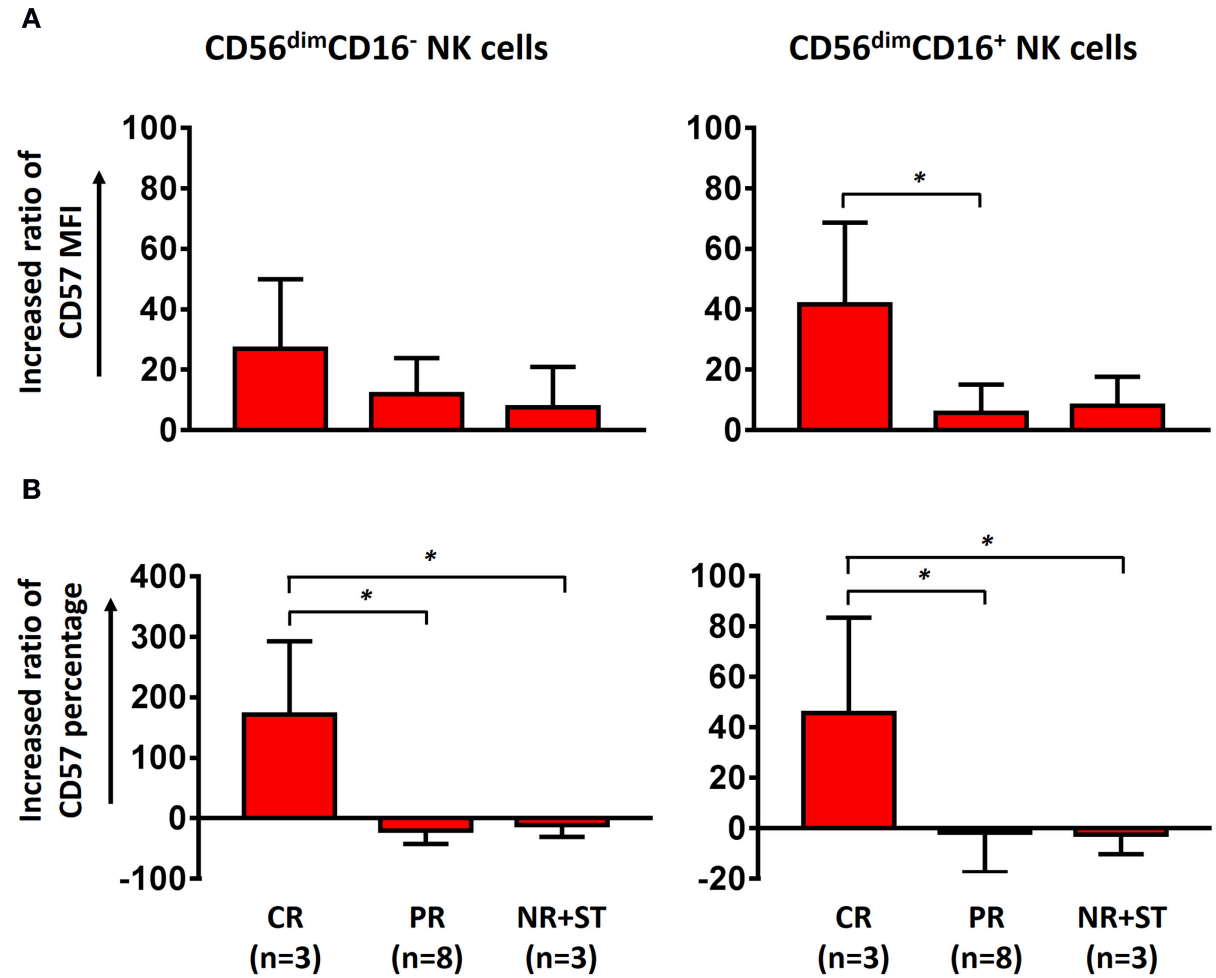
Effect of ECP on maturation of CD56^dim^ NK cells. Both the MFI of CD57 on CD56^dim^ NK cells **(A)** and the frequency of CD57^+^CD56^dim^ NK cells **(B)** were upregulated in aGVHD patients with CR (*n* = 3, excluding patient #8 due to the limited cell number of sample) but not in patients with PR (*n* = 8) and NR+ST (*n* = 3, excluding patient #5 due to the limited cell number of sample) after ECP treatment. The increasing ratio of the percentage was been calculated as followed: [(percentage of T2 – percentage of T1) × 100]/percentage of T1. The increasing ratio of the MFI was calculated as followed: [(MFI of T2 – MFI of T1) × 100]/MFI of T1. ^*^p < 0.05.

After ECP treatment, the quality (Figure 3A) and quantity (Figure 3B) of the maturation marker CD57 were upregulated in both CD56^dim^CD16^−^ and CD56^dim^CD16^+^ subsets in patients with aGVHD achieving CR as compared to patients with PR, NR, and ST, which suggests that ECP can promote the maturation of CD56^dim^CD16^−^ and CD56^dim^CD16^+^ NK cells in patients with aGVHD with favorable outcome.

### Shifting the Quality of NK Cells From Cytotoxicity to Regulation by ECP Treatment

**Figure 4:**
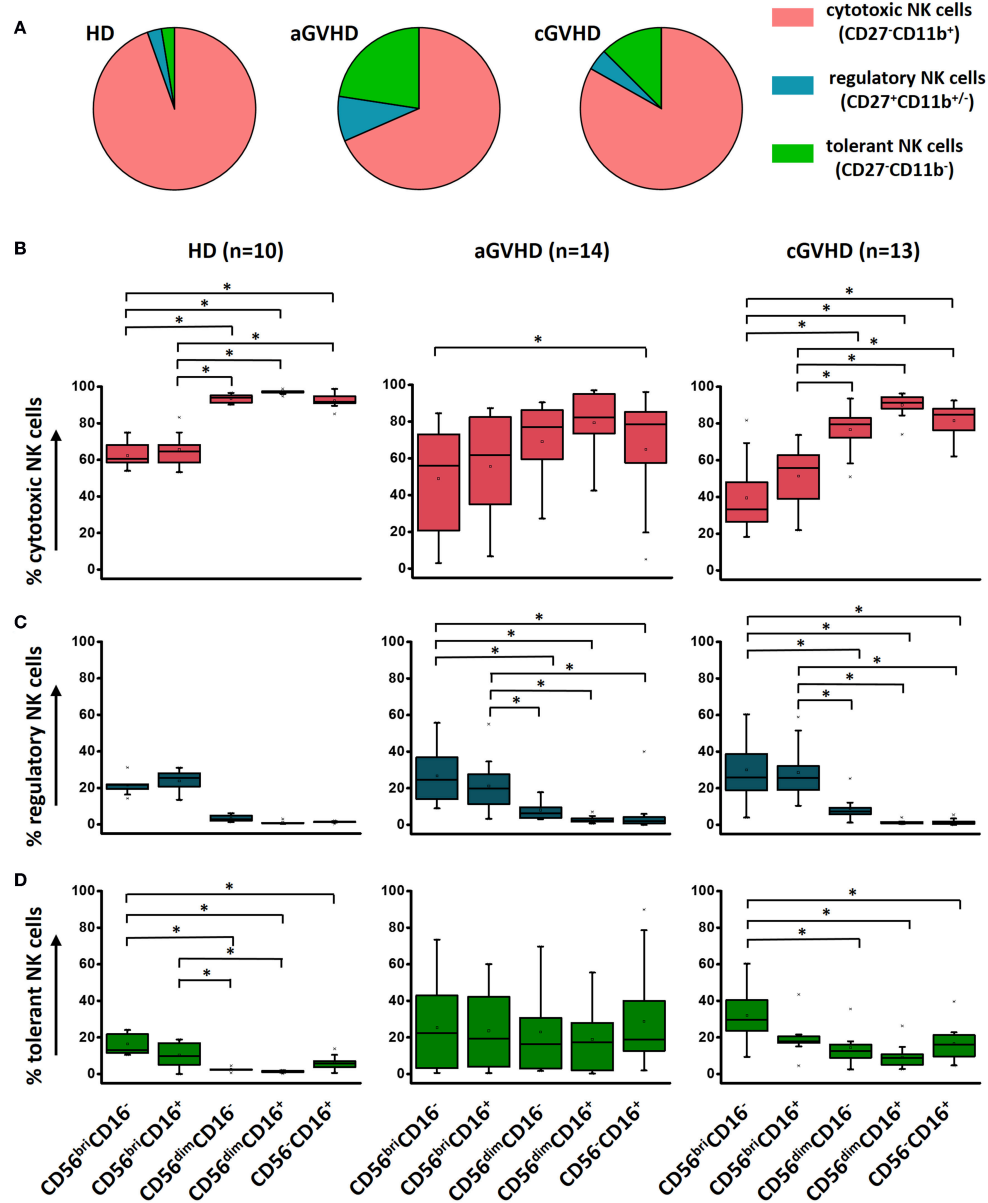
Characterization of functional NK subsets. Based on the expression of CD11b and CD27, NK cells could be defined as three functional subsets: CD11b^+^CD27^−^ cytotoxic NK cells, CD11b^+/−^CD27^+^ regulatory NK cells and CD11b^−^CD27^−^ tolerant NK cells. Although the frequency of these three subsets is different among HDs (*n* = 10), aGVHD (*n* = 14, excluding patient #5 and #8 due to the limited cell number of samples) and cGVHD (*n* = 13, excluding patient #17, #18, #21, #22, and #25 due to the limited cell number of samples) groups **(A)**, the component of cytotoxic **(B)**, and regulatory **(C)** NK cells is similar in five subpopulations among three different groups except the tolerant NK cells **(D)**. ^*^p < 0.05.

Functional NK cell populations, cytotoxic, regulatory, and tolerant NK cells, were defined in our study based on the relative expression of CD27 and CD11b, as shown in a previous study (23). The components of regulatory NK cells (CD27^+^CD11b^+/−^) and tolerant NK cells (CD27^−^CD11b^−^) were significantly increased in patients with GVHD compared to HDs (Figure 4A). However, the distribution of these three functional NK subsets within these five different NK subcategories was similar among HDs, aGVHD, and cGVHD patients (Figures 4B–D) with exception of an increased tolerant NK cell population in the aGVHD group (Figure 4D).

**Figure 5:**
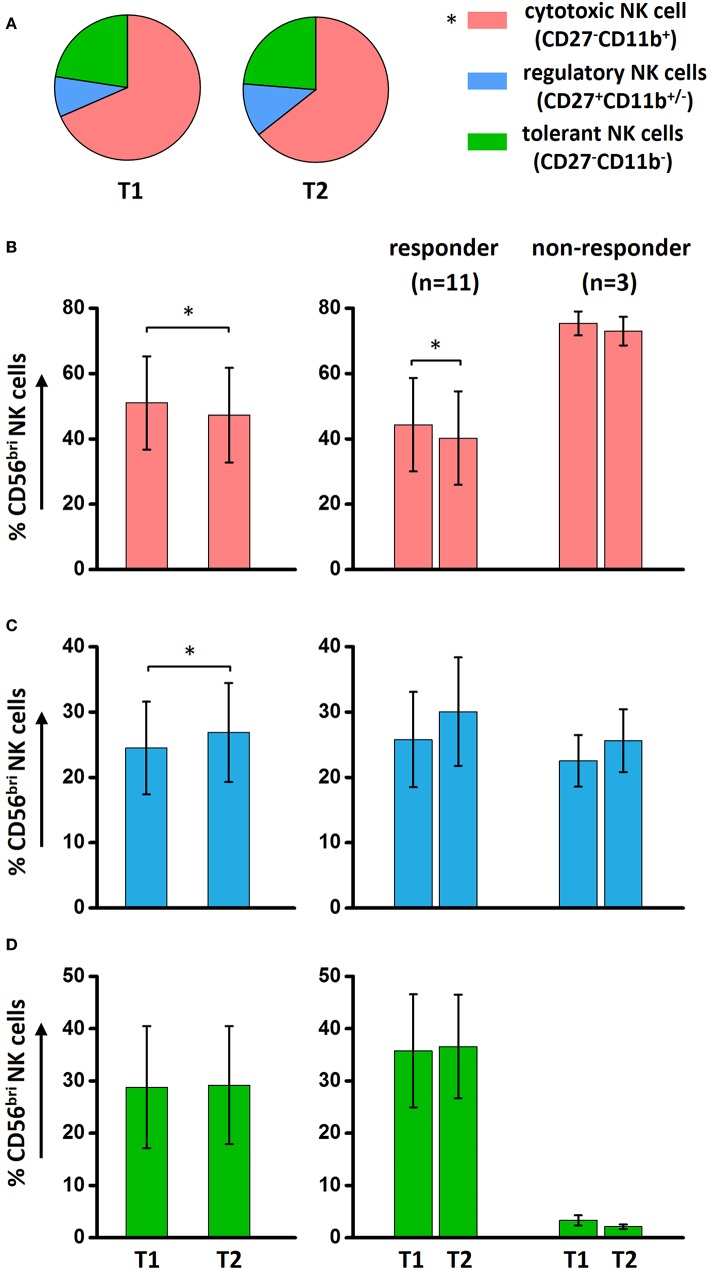
Effect of ECP on functional NK subsets. ECP could reduce significantly the total cytotoxic NK cells while keeping the total regulatory and tolerant NK cells stable in aGVHD patients **(A)**. However, within the CD56^bri^ NK subsets, a dramatically decrease of cytotoxic NK cells **(B)** in conjunction with an increase of regulatory NK cells **(C)** has been observed in aGVHD patient with response under ECP therapy. **(D)** The effect of ECP therapy has not been observed on CD56^bri^ tolerant NK cells. The responder group includes the patients with CR (*n* = 3, excluding patient #8 due to the limited cell number of sample) and patients with PR (*n* = 8). The non-responder group includes the patients with NR (*n* = 1, excluding patient #5 due to the limited cell number of sample) and patients with ST (*n* = 2). ^*^p < 0.05.

To investigate whether the functional NK cell subsets are influenced by ECP treatment, a comprehensive analysis was performed. Our results show that a significant decrease of cytotoxic CD27^−^CD11b^+^ NK cells was observed in aGVHD patients after ECP therapy (Supplementary Figure 2A), caused by the dramatic reduction of cytotoxic NK cells within the CD56^bri^ NK cell populations (Supplementary Figure 2B, Figures 5A,B). Furthermore, we observed a significant downregulation only in aGVHD responders while not in non-responders (Figure 5B). This confirms that this general reduction correlates with ECP response. In parallel, regulatory NK cells within CD56^bri^ NK cell subsets were significantly increased by ECP therapy (Figure 5C, Supplementary Figure 2C). However, there were no significant changes of tolerant NK cells (Figure 5D, Supplementary Figure 2D). Collectively, our data suggest that ECP therapy could shift the quality of CD56^bri^ NK cells from cytotoxic to regulatory NK cells.

### Intact Anti-Viral/Tumor Capacity of NK Cells Under ECP Treatment

**Figure 6:**
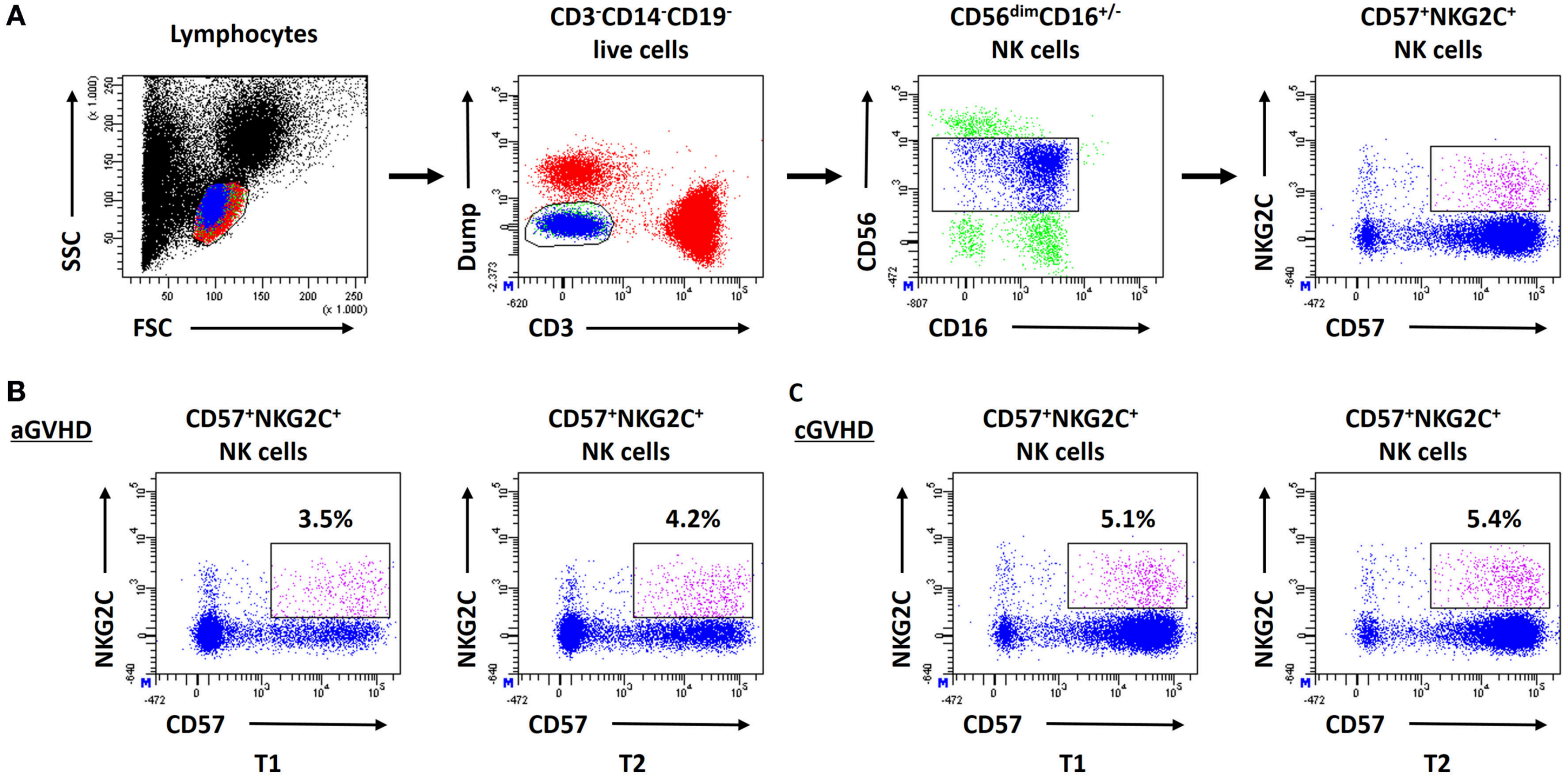
Effect of ECP on CD56^dim^CD57^+^NKG2C^+^ NK cells. **(A)** shows the analysis strategy of CD56^dim^CD57^+^NKG2C^+^ NK cells. This specialized anti-viral/relapse subset could be preserved after long-term ECP treatment in both aGVHD **(B)** and cGVHD **(C)** patients.

To determine the influence of ECP therapy on the anti-viral/tumor capacity of NK cells, a specialized anti-viral/tumor population, CD56^dim^CD57^+^NKG2C^+^ NK cells (24), as well as the quality and quantity of NK activity were monitored during ECP treatment. CD56^dim^CD57^+^NKG2C^+^ NK cells were identified in our study following the strategy as shown in Figure 6A. A stable frequency of CD56^dim^CD57^+^NKG2C^+^ NK cells during ECP therapy was observed in both aGVHD (Figure 6B) and cGVHD patients (Figure 6C).

**Figure 7:**
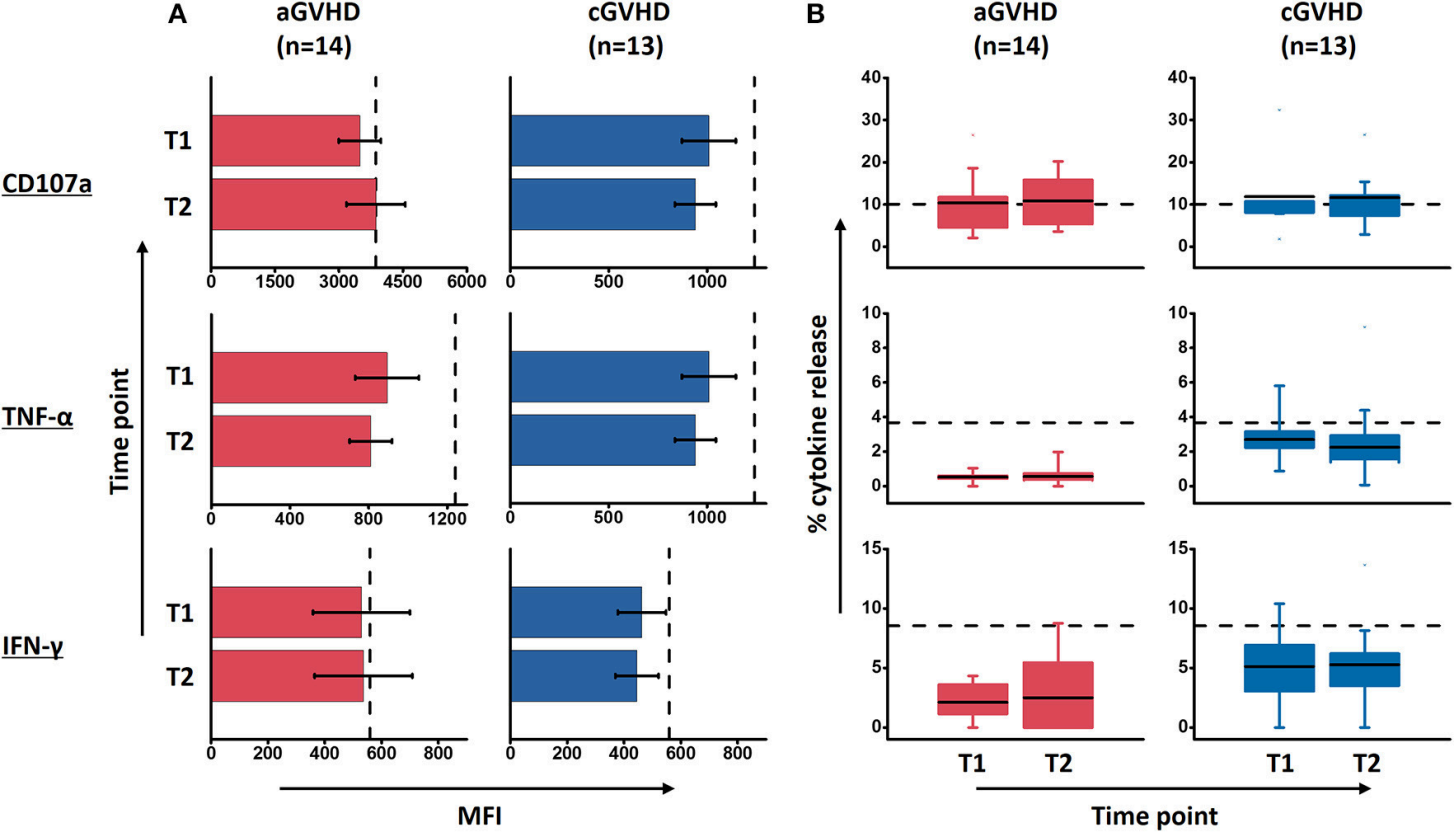
Effect of ECP on the quality and quantity of NK cell function upon K562 stimulation. ECP therapy has no negative effect on the quality of NK cell function in terms of MFI of marker expression **(A)** as well as the quantity of CD107 expression and the cytokine release by NK cells **(B)**. Fourteen patients with aGVHD, excluding patient #5 and #8 due to the limited cell number of samples, and 13 patients with cGVHD, excluding patient #17, #18, #21, #22, and #25 due to the limited cell number of samples, were analyzed. The dashed lines represent the mean value of 10 healthy donor controls.

**Figure 8:**
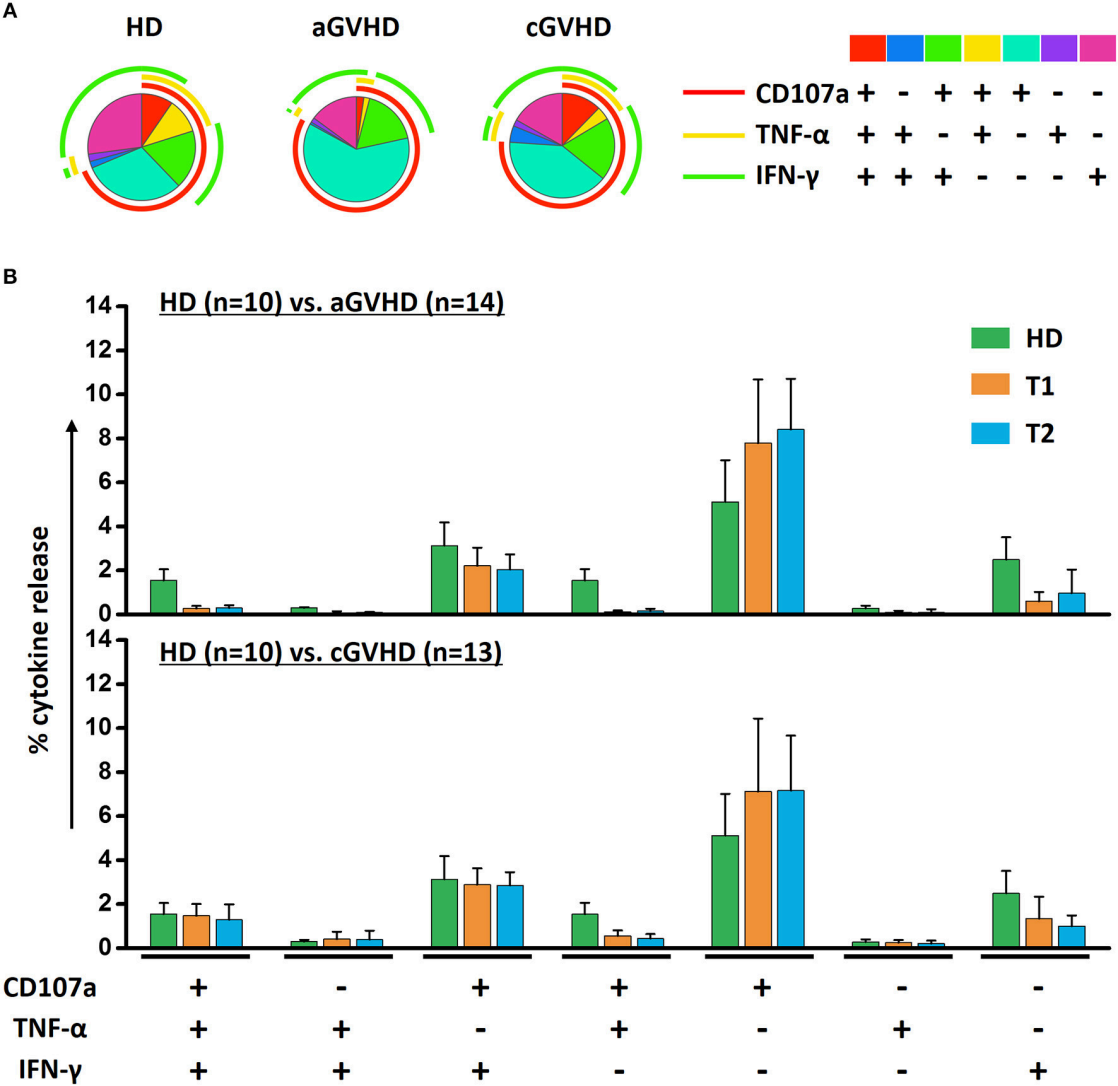
Effects of ECP on the multifunctional NK cells. The polyfunctional NK cells were analyzed using Boolean gating strategy. The pattern of multifunctional NK cells was different among HDs (*n* = 10), aGVHD (*n* = 14, excluding patient #5 and #8 due to the limited cell number of samples) and cGVHD (*n* = 13, excluding patient #17, #18, #21, #22, and #25 due to the limited cell number of samples) patients **(A)**. After long-term ECP treatment, the polyfunctional NK cells could keep stable **(B)**.

The mean fluorescence intensity (MFI) reflecting the cytokine release on a per-cell basis has been the subject of this study, with further interest due to the quality of NK cell response. There was no significant alteration of the MFI of CD107a, TNF-α and IFN-γ during ECP therapy in our study (Figure 7A). The frequency of CD107a expression and the cytokine release by NK cells upon K562 stimulation *in vitro* were maintained as well (Figure 7B). Of note, even though the multifunctional NK cells which are associated with enhanced effector function showed different patterns among HDs, aGVHD and cGVHD patients (Figure 8A), the multifunctionality of NK cells was constant during ECP therapy (Figure 8B).

**Figure 9:**
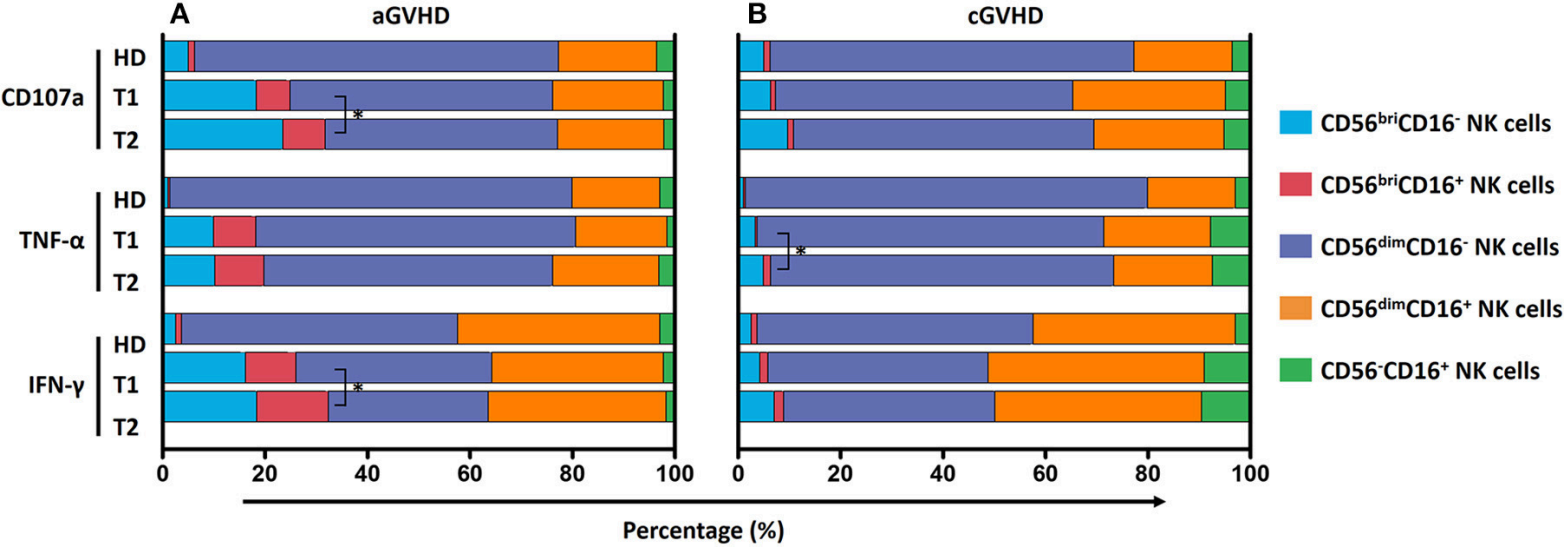
Effect of ECP on the contribution of NK subsets to GVL effect. The contribution of five different NK subpopulations with the respect to CD107a expression, TNF-α and IFN-γ secretion was investigated in both aGVHD (*n* = 14, excluding patient #5 and #8 due to the limited cell number of samples) **(A)** and cGVHD patients (*n* = 13, excluding patient #17, #18, #21, #22, and #25 due to the limited cell number of samples) **(B)**. ECP therapy could improve CD107a expression and IFN-γ secretion by CD56^bri^CD16^+/−^ NK cells in aGVHD patients **(A)**, while improve TNF-α secretion by CD56^bri^CD16^+/−^ NK cells in cGVHD patients **(B)**. ^*^p < 0.05.

Since five different NK cell subsets were defined in our study, we compared the contribution of these five subsets to the anti-tumor function and further assessed whether it would be influenced by ECP therapy. We found a significant improvement of CD107a expression and IFN-γ release by CD56^bri^ NK cells in the aGVHD cohort (Figure 9A) as well as secretion of TNF-α by CD56^bri^ NK cells in cGVHD patients (Figure 9B). Apparently, our data suggest that ECP could maintain or even improve the functionality of NK cells with respect to the anti-viral/tumor capabilities via preserving the frequency of CD56^dim^CD57^+^NKG2C^+^ NK cells and keeping the quality and quantity of the cytokine profile of the NK cells.

### Preserving Proliferative Function of NK and T Cells After ECP Treatment

**Figure 10:**
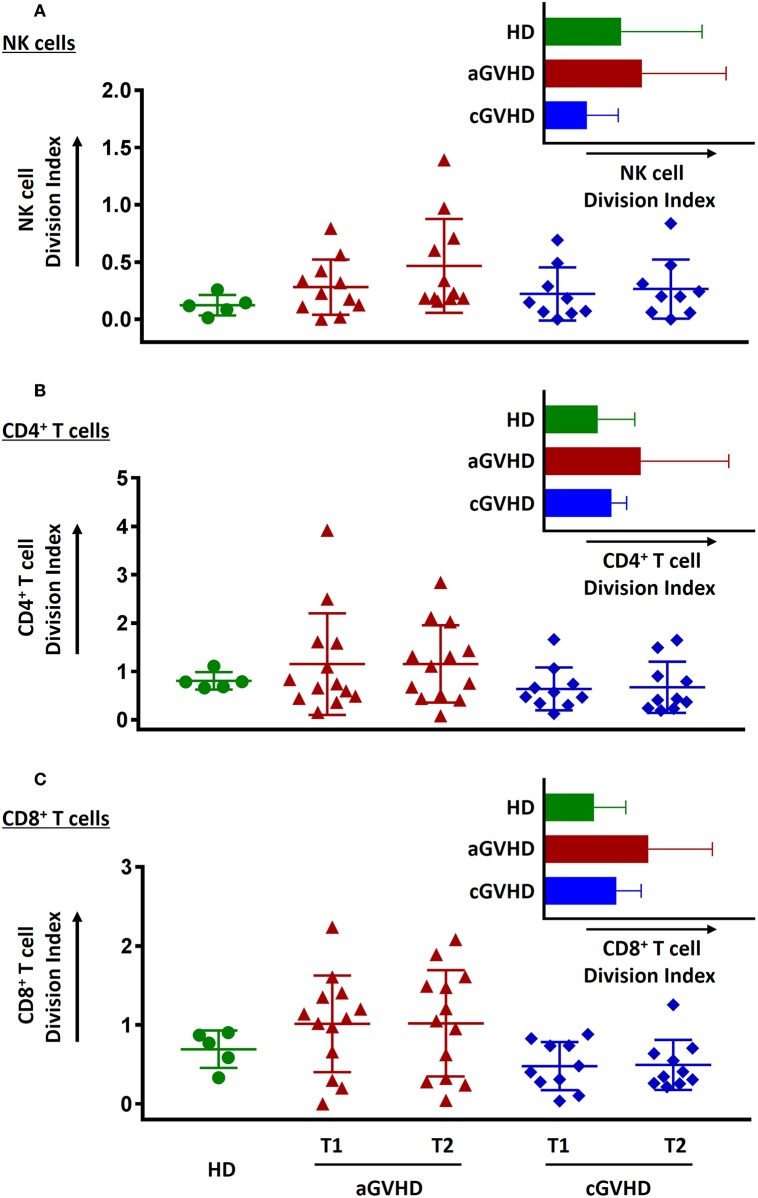
Effect of ECP on cell proliferative function. The proliferative capacity of NK cells **(A)**, CD4^+^ T cells **(B)**, and CD8^+^ T cells **(C)** was evaluated using CFSE staining. None of them could be hampered by ECP therapy. All the proliferation assays were performed with 11 samples from aGVHD patients, excluding the samples from patient #5, #6, #8, #10, and #11 due to the limited cell number of samples, nine samples from cGVHD patients, excluding the samples from patient #17, #18, #19, #21, #22, #25, #26, #27, and #31 due to the limited cell number of samples, and five HDs.

Proliferative capacity as an important cell function was evaluated in our study. Freshly thawed PBMCs stained with CFSE were stimulated either by IL-15 or SEB to determine the proliferative capabilities of the NK cells and T cells, respectively. NK cells (Figure 10A), CD4^+^ T cells (Figure 10B), and CD8^+^ T cells (Figure 10C) from aGVHD patients had greater proliferative capacity than HDs and patients with cGVHD. In contrast, NK cells but not CD4^+^ and CD8^+^ T cells from cGVHD patients showed a lower proliferative potential than HDs (Figure 10). Moreover, ECP therapy did not hamper neither NK cell (Figure 10A) nor T cell proliferative capacity (Figures 10B, C).

## Discussion

In corollary to our previous studies (5, 25), we further focused on NK cells to investigate their role in ECP therapy for GVHD patients. In the current study, we could show that (a) the heterogeneous NK cell population presents distinct patterns among HDs and patients with aGVHD and cGVHD. (b) A higher frequency of CD56^bri^ NK subset with stronger NKG2D and CD62L expression was found in patients with aGVHD when compared to those with cGVHD and HDs. In aGVHD patients achieving CR, ECP therapy could not only (c) decrease significantly CD56^bri^CD16^−^ NK cells with shifting the property from cytotoxic to regulatory NK subsets but also (d) mature the CD56^dim^ NK cells through up-regulation of CD57. Most important, ECP could keep the intact anti-viral and anti-leukemic effects via (e) maintaining specialized anti-viral/leukemic CD57^+^NKG2C^+^CD56^dim^ NK cells as well as (f) preserving the quantity and quality in terms of the MFI of cytokines, polyfunctionality and proliferative capacity of NK cells under ECP treatment.

Immune reconstitution after allo-HSCT is a complex process. The unbalanced or the delayed and incomplete immune reconstitution could result in not only expansion of alloreactive T cells leading to GVHD but also in a higher incidence of opportunistic infections (5, 26). Thus, therapeutic strategies to accelerate immune reconstitution after allo-HSCT might be a reasonable way for both GVHD treatment and prevention of infections.

ECP therapy with promising clinical outcome constitutes an effective immunomodulatory therapy for GVHD. In our series, 75% of aGVHD and 78% of cGVHD patients obtained clinical response. Notably, our previous studies indicated that ECP therapy could support the immune recovery after allo-HSCT (5, 25). Therefore, ECP represents an attractive strategy to treat GVHD.

NK cells are known to be the first and predominant donor-derived lymphocytes to reconstitute after allo-HSCT (6, 12, 24, 27). The role of NK cells in the development and the prevention of GVHD is paradoxical. NK cells might enhance inflammation by secretion of TNF-α and IFN-γ and thus promote GVHD but on the other hand sustain homeostasis through targeted killing of activated alloreactive T cells and antigen presenting cells to control GVHD (28, 29). The heterogeneity within the NK subset might further contribute to these conflicting effects during GVHD. According to the expression of CD56 and CD16, we identified five different NK cell populations with different immunophenotype and function. Moreover, our results show that the composition of NK cells is explicitly divergent among HDs, aGVHD, and cGVHD patients, suggesting that different NK subsets are involved in the pathogenesis of aGVHD and cGVHD.

In patients suffering from steroid refractory/resistant aGVHD, we observed a dramatically increased in the CD56^bri^ NK subset compared to HDs and cGVHD patients, where CD56^dim^ NK cells constitute the majority of the NK cells. The origin of CD56^bri^ NK cells is still a hot debate. CD56^bri^ NK cells descending from common lymphoid progenitors or common myeloid progenitors are considered to be the immature precursors of CD56^dim^ NK cells in a linear-differentiation model (30, 31). In the early period post-HSCT, a high frequency of CD56^bri^ NK cells reflects a better immune reconstitution (9, 12, 32). This provides a biological explanation for a previous report indicating that ECP therapy could increase the CD56^bri^ NK cells in responding GVHD patients during the early ECP treatment phase (14). On the other hand, CD56^dim^ NK cells could upregulate CD56 after activation, suggesting a proportion of CD56^bri^ NK cells might be activated NK cells rather than immature precursors (31, 33). Both theories regarding the origin of CD56^bri^ NK cells are supported by our observation in aGVHD patients that CD56^bri^ NK cells express high levels of immature marker CD62L (27, 34) and also activation receptor NKG2D (35).

CD56^bri^ NK cells are believed to have a strong cytokine production capacity with a weakly cytolytic potential (12, 36, 37). Since the NKG2D ligands, MHC class I-related Chains A and B (MICA and MICB) as well as UL-16 binding proteins (ULBP1-4), are extensively induced in skin, gut, and liver during aGVHD, these ligands could recruit the activated cytokine-producing NKG2D^+^CD56^bri^ NK cells into the target tissues to directly damage cells (38). In turn, the proinflammatory cytokines released by the injured tissue could cause increased secretion of TNF-α and IFN-γ *in situ* by activated CD56^bri^ NK cells creating an amplification loop that leads to further deterioration of GVHD by direct cell damage or indirect T cell-mediated tissue damage (18, 39, 40). This might explain our observation of a dramatic reduction of CD56^bri^ NK cells with decreasing NKG2D expression in aGVHD patients undergoing ECP therapy in association with a favorable clinical outcome.

This observation seems to be in conflict with a recent ECP study performed in GVHD patients reporting that an early increase of CD56^bri^ NK cells is a dominant effect and predicts response to ECP (14). However, the discrepancy between this study and our data might be explained by a longer immunomonitoring time span of patients under ECP therapy in our study. As described, CD56^dim^ NK cells display a mature phenotype and majorly contribute to immune defense and GVHD-reducing effect in contrast to CD56^bri^ subsets (24, 41, 42). Indeed, a higher expression of differentiation markers CD11b and CD57 on CD56^dim^ NK cells than on CD56^bri^ NK cells confirmed their mature phenotype in our study. Recently, preclinical data showed that alloreactive T cells could impair the reconstitution and maturation of donor NK cells through competition for the critical survival/differentiation cytokine IL-15, switching NK cells toward early immature NK cells that are known to survive at low levels of IL-15 (32). Reasonably, we assume that priming of CD56^bri^ NK cells by ECP therapy in the early treatment phase is a prerequisite for sequential steps of NK cell differentiation. Apart from this, our previous study indicated that ECP promotes the NK cell differentiation via losing immature receptor CD62L (5). In addition, the significant increase of the density of CD57 on CD56^dim^ NK cells and the frequency of CD57^+^CD56^dim^ NK cells further support our theory of NK cell differentiation by ECP, since acquisition of CD57 on NK cells is an irreversible process for NK cell maturation (43–46).

Most interesting data emerge from the dissection of the components of NK cells based on the expression of CD11b and CD27. Significant increases of regulatory (CD27^+^CD11b^+/−^) and tolerant (CD27^−^CD11b^−^) NK cells were observed in GVHD patients, suggesting NK cells could have immunoregulatory properties under certain conditions. Those regulatory NK cells could control the inflammation via either induction of other regulatory cells such as regulatory T cells, tolerogenic dendritic cells and monocytes or via suppression of Th17 cells (23, 47). This pleiotropic nature of NK cells might be likely responsible for the variable and even conflicting roles in the development of GVHD. Of note, ECP could shift the NK cells from a cytotoxic to a regulatory/tolerant phenotype, especially, within the CD56^bri^ subset. This shaping effect might partly contribute to the induction of NK cells (48) and CD56^bri^ NK cells (14) by ECP therapy as well.

In the case of cGVHD, CD56^−^ NK cells stand out as a signature NK subset. Previous studies have shown that the existence of CD56^−^ NK cells is associated with chronic viral infection e.g., human immunodeficiency virus and hepatitis C, where NK cells display an impaired functionality with an exhausted phenotype (37, 49–53). In line with these studies, CD56^−^CD16^+^ NK cells in cGVHD patients highly express terminally differentiated markers and display a low capacity of cytotoxicity and cytokine release upon K562 stimulation. However, the patients with higher frequency of CD56^−^ NK cells had no viral infection nor virus reactivation, suggesting that cGVHD with a persistent inflammation could drive mature NK cells toward the unfunctional CD56^−^ NK cells as well. However, we did not observe any effects on CD56^−^ NK subset by ECP therapy.

Although ECP therapy could induce immune tolerance and rebalance the immune system, there is no clinical reports showing that ECP is associated with an increased risk of infection and relapse of primary disease (5, 54, 55). In our previous study, we for the first time proved that ECP therapy preserves immunity against infections and the graft vs. leukemia (GVL) effect on the cellular level (5). Based on these findings, we further investigated whether ECP could influence the function of NK cells, since NK cells mediate important innate immunity that bridges the T-cell-deficient period after HSCT in order to control the viral infections and eliminate the residual malignant cells (32, 56, 57).

A specialized subset of NK cells with a CD56^dim^CD57^+^NKG2C^+^ phenotype that is highly associated with anti-viral and GVL effect was monitored in the current study. ECP had no negative influence on the frequency of this subset. Consequently, CD56^dim^CD57^+^NKG2C^+^ NK cells could still functionally mediate the GVL effect either through secreting TNF-α and IFN-γ or via NKG2C binding to HLA-E (24). Our data further confirmed that the production of TNF-α and IFN-γ by NK cells in response to K562 myeloid leukemia cells was not affected by ECP treatment. Similarly, lysosomal-associated membrane protein-1 (CD107a), a sensitive marker of NK activity (58), was stably expressed by NK cells upon K562 stimulation under ECP therapy.

The magnitude of immune response is a fundamental characteristic of NK cell-mediated immune defense. However, the quality of NK cell responses is more crucial for determining their functionality. With respect to this, the MFI reflecting the cytokine release on a per-cell basis and the polyfunctionality of NK cells associated with enhanced effector function were assessed in GVHD patients under ECP therapy (59). No significant changes were observed. Notably, ECP could even enhance the NK cell-mediated GVL effect via increase of cytokine release by CD56^bri^ NK cells. Furthermore, the proliferation of NK cells providing an expanded pool of effector cells against the pathogens was not hampered by ECP therapy as well. Summing up, our data suggest that ECP does not comprise the quantity and quality of NK activity for control of virus reactivation and anti-tumor immunity post-transplant.

Importantly, immunosuppressive medications should be considered for changes of different cell populations and markers as well. In our study, the major change in immunosuppressive therapy next to ECP treatment was the reduction of steroids. This reduction however was not associated with similar changes of cell subsets among patients with different clinical responses. Therefore, we assume that the reduction of steroids might not contribute to the changes of the NK subsets.

**Figure 11:**
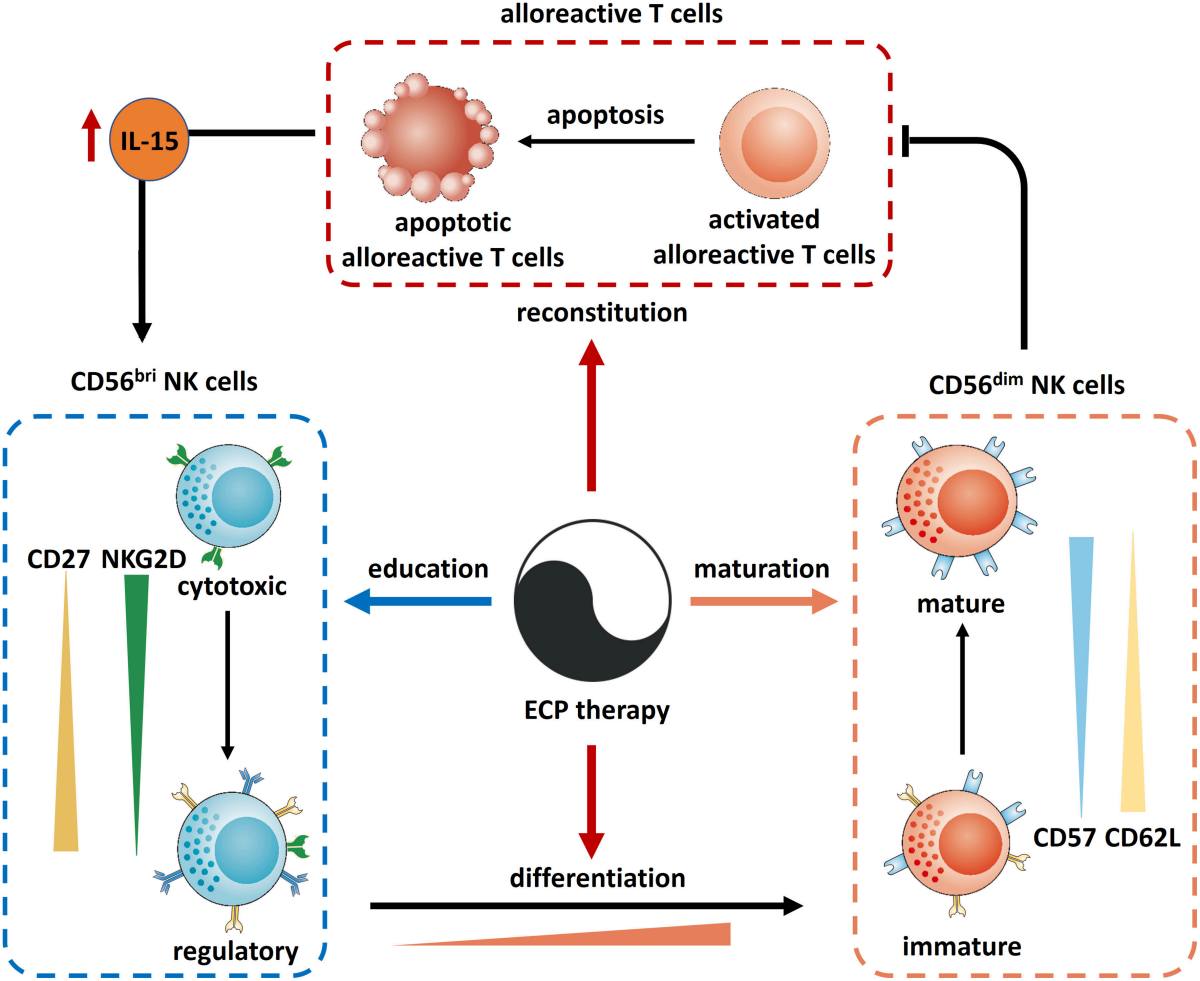
Mechanism of action of ECP on NK cells in GVHD. Reconstitution: ECP could induce the apoptosis of alloreactive T cells resulting in an elevation of IL-15 which recoveries the immune reconstitution. Differentiation: IL-15 could also promote the differentiation of NK cells from CD56^bir^ subset to CD56^dim^ subset. In turn, CD56^dim^ NK cells could kill the alloreactive T cells. Maturation: CD56^dim^ NK cells could be matured through ECP therapy via upregulation of CD57 and downregulation of CD62L. Education: ECP could educate CD56^bri^ NK cells by shifting their quality from a cytotoxic to a regulatory function.

Incorporating the results from former studies, the underling mechanisms behind the effects of ECP on NK cells are summarized in Figure 11. ECP therapy could directly induce alloreactive T cell apoptosis which results in sparing of IL-15. Consequently, this promotes not only the recovery of immune reconstitution with an increase of CD56^bri^ NK cells but also a differentiation of NK cells from an immature phenotype CD56^bri^ to a mature state CD56^dim^ followed by further maturation of CD56^dim^ NK cells. Moreover, ECP could educate CD56^bri^ NK cells by shifting their quality from a cytotoxic to a regulatory function.

In conclusion, ECP therapy represents a safe and effective immunomodulatory treatment for GVHD patients through its effects on reconstitution, differentiation, maturation and education of NK cells.

## Ethics Statement

The study was approved by the local Ethical Committees and all patients signed consent forms before treatment.

## Author Contributions

AS, MS, LW, and MN designed the research. MN, LW, and MY performed the experiments. AS, M-LS, TL, UH, SS, PW, WK, LS, MS, and RY treated the patients. KB collected and shipped the samples. MN and LW acquired and analyzed the data. MS, AS, MN, LW, AH-K, BC, PD, CM-T, RY, AN, BN, and WK discussed the organization of the manuscript. LW and MN wrote the manuscript. All authors critically reviewed the manuscript. MS, AS, PD, CM-T, and AN edited the manuscript. AS, MS, and LW supervised the work.

### Conflict of Interest Statement

Therakos Mallinckrodt gave a financial support to AS and MS for the documentation of the clinical course and for the analysis of immune cells of the patients, PW has Honoraria and membership on Advisory Boards for Sanofi-Aventis. The remaining authors declare that the research was conducted in the absence of any commercial or financial relationships that could be construed as a potential conflict of interest.

## Supplementary Material

Supplementary Table 1 Antibody list.

Supplementary Figure 1 Effect of ECP on CD56briCD16- NK subsets in aGVHD patients without CR. The dramatic reduction of CD56briCD16- NK subset by ECP therapy could not be observed in patients with PR (*n* = 8), NR (*n* = 1), and ST (*n* = 2). Dashed line represents the corresponding median value of frequencies observed in 10 healthy donors.

Supplementary Figure 2 Effect of ECP on functional NK subsets. **(A)** The effects of ECP on the functional NK cell subsets were evaluated in both aGVHD (left panel) and cGVHD patients (right panel). A significant downregulation of the frequency of cytotoxic NK cells in conjunction with a slight upregulation of regulatory and tolerant NK cells was observed in aGVHD patients. The changes of cytotoxic, regulatory and tolerant NK cells within five different NK subpopulations by ECP have be shown in **(B–D)**, respectively.
