# Supplementary material for: Shaping of CD56bri Natural Killer Cells in Patients With Steroid-Refractory/Resistant Acute Graft-vs.-Host Disease via Extracorporeal Photopheresis
Source: Front Immunol. 2019 Mar 20;10:547. doi: 10.3389/fimmu.2019.00547 (PMC6436423; doi:10.3389/fimmu.2019.00547)
Supplement: Supplementary Table 1 — Antibody list. [file Table_1.docx]

Supplementary Material

Shaping of CD56^bri^ natural killer cells in patients with steroid-refractory/resistant acute graft-versus-host disease via extracorporeal photopheresis

Ming Ni^1,2Ϯ^, Lei Wang^1Ϯ^, Mingya Yang^1^, Brigitte Neuber^1^, Leopold Sellner^1^, Angela Hückelhoven-Krauss^1^, Maria-Luisa Schubert^1^, Thomas Luft^1^, Ute Hegenbart^1^, Stefan Schönland^1^, Patrick Wuchter^3^, Bao-an Chen^4^, Volker Eckstein^1^, William Krüger^5^, Ronit Yerushalmi^6^, Katia Beider^6^, Arnon Nagler^6^, Carsten Müller-Tidow^1^, Peter Dreger^1^, Michael Schmitt^1^, Anita Schmitt^1*^

Ϯ Both authors contributed equally to this work.

^1^Department of Internal Medicine V, University Clinic Heidelberg, Heidelberg, Germany.

^2^Department of Hematology, the Affiliated Hospital of Guizhou Medical University, Guizhou, China.

^3^German Red Cross Blood Service, Medical Faculty Mannheim, Institute of Transfusion Medicine and Immunology Mannheim, Mannheim, Germany.

^4^Department of Hematology, Zhongda Hospital, Southeast University, Nanjing, China.

^5^Department of Internal Medicine C, Haematology, Oncology, Stem Cell Transplantation, Palliative Care, University Clinic Greifswald, Greifswald, Germany.

^6^Hematology Division, Chaim Sheba Medical Center, Tel Hashomer, Israel.

***Correspondence:**PD Dr. med. Anita Schmitt
[anita.schmitt@med.uni-heidelberg.de](mailto:anita.schmitt@med.uni-heidelberg.de)

# Supplementary Table 1. Antibody list.

| **Name** | **Dye** | **Isotype** | **Clone** | **V_Working_ (μl)** | **Company** | **Cat.NO.** |
| --- | --- | --- | --- | --- | --- | --- |
| CD3 | PE/Dazzle 594 | Mouse IgG2a, κ | HIT3a | 2 | Biolegend | 300336 |
| CD3 | BV510 | Mouse IgG1, κ | UCHT1 | 1 | Biolegend | 300448 |
| CD4 | PE | Mouse IgG2b, κ | OKT4 | 1 | Biolegend | 317410 |
| CD8 | PerCP | Mouse IgG1, κ | SK1 | 0.5 | Biolegend | 344708 |
| CD11b | BV510 | Mouse IgG1, κ | ICRF44 | 2 | BD | 563088 |
| CD14 | APC-eFluor780 | Mouse IgG1, κ | 61D3 | 2 | Thermo Fisher | 47-0149-42 |
| CD14 | PerCP | Mouse IgG1, κ | HCD14 | 2 | Biolegend | 325632 |
| CD16 | APC-Cy7 | Mouse IgG1, κ | 3G8 | 4 | Biolegend | 302018 |
| CD19 | PerCP | Mouse IgG1, κ | HIB19 | 2 | Biolegend | 302228 |
| CD19 | APC-Cy7 | Mouse IgG1, κ | HIB19 | 2 | Biolegend | 302218 |
| CD27 | PE-Cy7 | Mouse IgG1, κ | M-T271 | 2 | Biolegend | 356412 |
| CD56 | APC | Mouse IgG1, κ | HCD56 | 3 | Biolegend | 318309 |
| CD57 | Pacific Blue | Mouse IgM, κ | HCD57 | 1 | Biolegend | 322316 |
| CD62L | eFluor 450 | Mouse IgG1, κ | DREG56 | 3 | Thermo Fisher | 48-0629-42 |
| CD107a | FITC | Mouse IgG1, κ | H4A3 | 10 | BD | 560949 |
| CD159c (NKG2C) | PE | Mouse IgG1, κ | 134591 | 5 | r&d systems | FAB138P |
| CD314 (NKG2D) | PE | Mouse IgG1, κ | 1D11 | 1 | BD | 557940 |
| IFN-γ | PE | Mouse IgG1, κ | B27 | 1 | BD | 554701 |
| TNF-α | BV421 | Mouse IgG1, κ | MAb11 | 1 | BD | 562783 |

Abbreviations: Vworking = working volume of the respective antibody; Cat. No. = catalogue number; PE = phycoerythrin; BV = Brilliant Violet; PerCP = peridinin chlorophyll; APC = allophycocyanin; APC-Cy7 = allophycocyanin-Cyanin 7; PE-Cy7 = phycoerythrin-Cyanin 7. IFN-γ = Interferon-γ; TNF-α = Tumor necrosis factor-α; FITC = Fluorescein isothiocyanate.
