# Supplementary figures and images for: Shaping of CD56bri Natural Killer Cells in Patients With Steroid-Refractory/Resistant Acute Graft-vs.-Host Disease via Extracorporeal Photopheresis
Source: Front Immunol. 2019 Mar 20;10:547. doi: 10.3389/fimmu.2019.00547 (PMC6436423; doi:10.3389/fimmu.2019.00547)

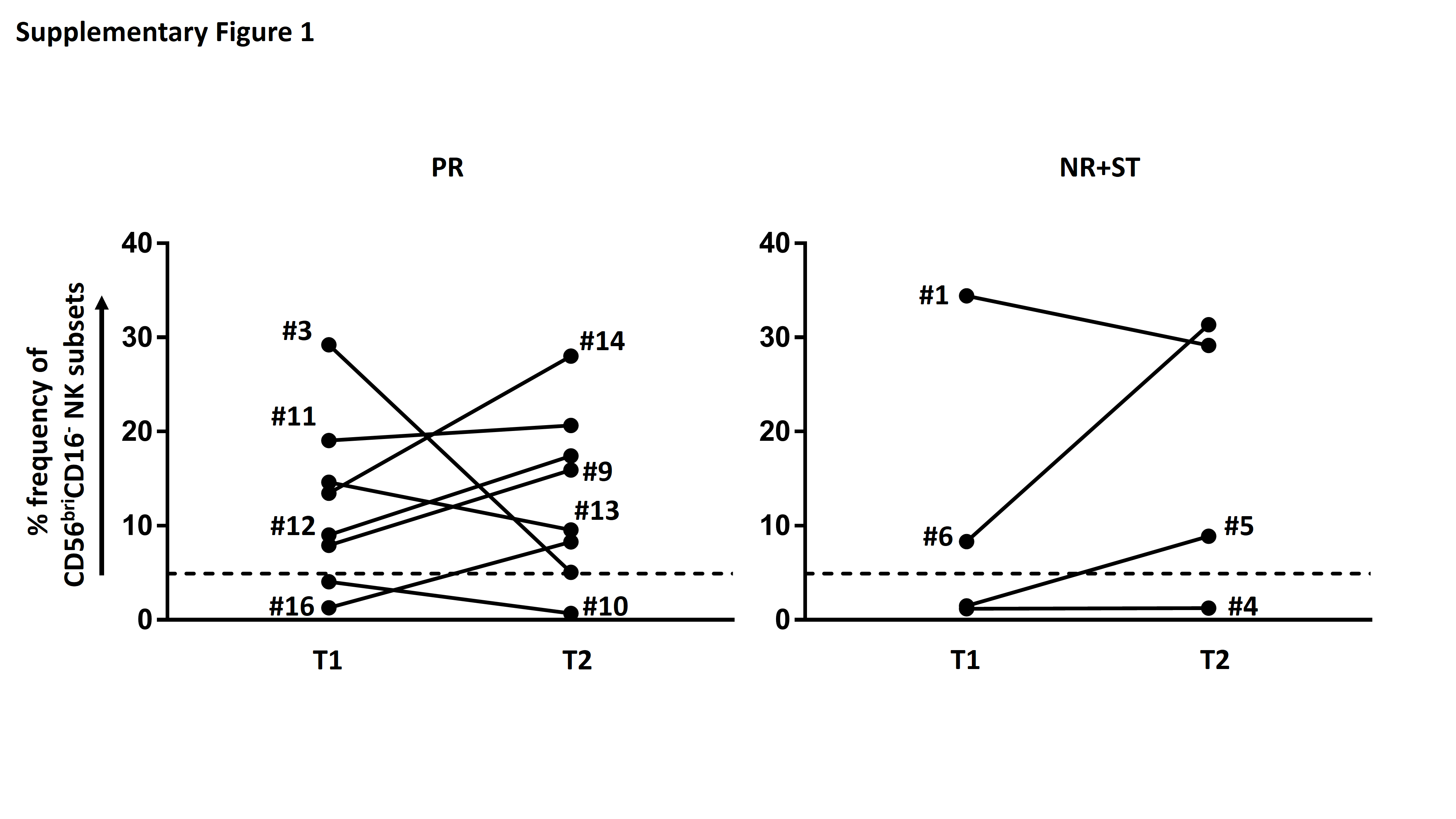

Supplement: Supplementary Figure 1 — Effect of ECP on CD56briCD16- NK subsets in aGVHD patients without CR. The dramatic reduction of CD56briCD16- NK subset by ECP therapy could not be observed in patients with PR (n = 8), NR (n = 1), and ST (n = 2). Dashed line represents the corresponding median value of frequencies observed in 10 healthy donors. [file Image_1.TIF]

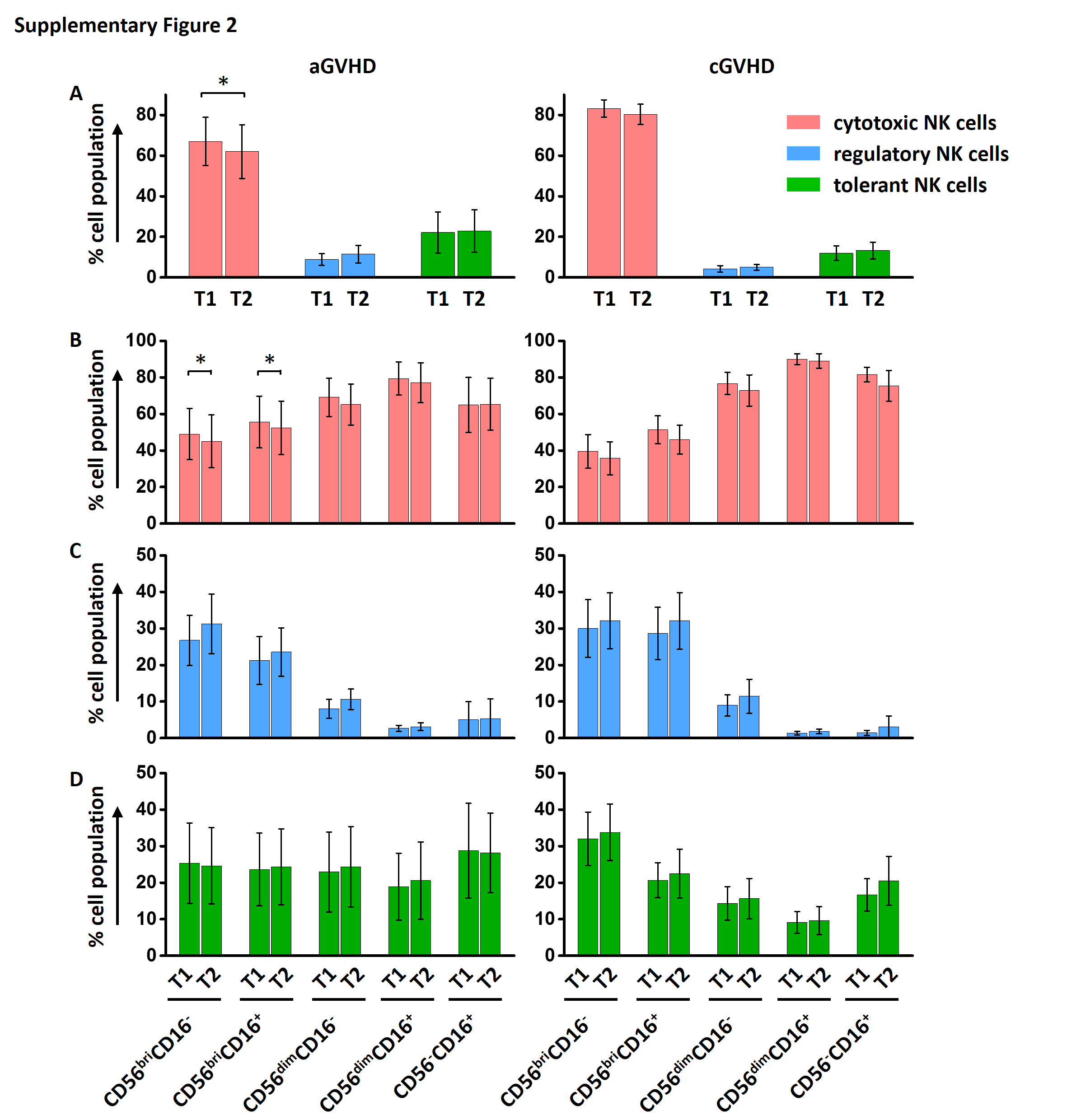

Supplement: Supplementary Figure 2 — Effect of ECP on functional NK subsets. (A) The effects of ECP on the functional NK cell subsets were evaluated in both aGVHD (left panel) and cGVHD patients (right panel). A significant downregulation of the frequency of cytotoxic NK cells in conjunction with a slight upregulation of regulatory and tolerant NK cells was observed in aGVHD patients. The changes of cytotoxic, regulatory and tolerant NK cells within five different NK subpopulations by ECP have be shown in (B–D), respectively. [file Image_2.tif]
